# Mobile Interventions Targeting Risky Drinking Among University Students: A Review

**DOI:** 10.1007/s40429-016-0099-6

**Published:** 2016-04-05

**Authors:** Anne H. Berman, Mikael Gajecki, Kristina Sinadinovic, Claes Andersson

**Affiliations:** Department of Clinical Neuroscience, Center for Psychiatric Research, Karolinska Institutet, Norra Stationsgatan 69, 7th floor, SE-113 64 Stockholm, Sweden; Stockholm Center for Dependency Disorders, Box 179 14, SE-118 95 Stockholm, Sweden; Department of Health and Society, Malmö University, SE-20506 Malmö, Sweden

**Keywords:** Review, Alcohol, Hazardous drinking, University/college students, Intervention, Text messages, Automated telephony, Smartphone apps

## Abstract

Mobile interventions based on text messages, automated telephone programs (interactive voice response (IVR)), and smartphone apps offer a new approach targeting hazardous alcohol use in university students. This review covers seven recent studies involving college or university students that evaluated intervention efficacy in comparison to controls: four using text messages, one using IVR, and two smartphone apps. Only the study evaluating IVR reported positive results for the primary outcome. Two of the text message studies reported positive results on secondary outcomes, while the other two reported no differences in comparison to control groups. For smartphone apps, one study reported positive results on secondary outcomes, while the other showed no differences in comparison to controls for a web-based app and negative results for a native app. Further development of mobile interventions is needed for this at-risk population, both in terms of intervention content and use of robust research designs.

## Introduction

Research has consistently shown that alcohol consumption and alcohol-related risks peak in emerging adulthood, mainly between 18 and 25 years [1]. About half of all young adults enter higher education [2], making college and university students an important target population for alcohol interventions. Indeed, college and university students seem to establish their future adult alcohol habits during their years of higher education [3]. Numerous factors influence this development, on individual, social, and environmental levels [4]. Considerable research has been conducted in order to identify effective ways of reducing hazardous drinking among university students, and several studies have demonstrated that effective intervention approaches exist, both at individual and group levels. These methods are well-documented internationally [5, 6].

Although student health services provide interventions for drinking problem, only a small percentage of students seek such help [7]. One effective intervention approach for university students involves brief motivational interventions (BMIs) [5, 6]. These interventions provide personalized feedback on individual drinking habits and their consequences, often based on self-monitoring. Also, students explore their motives for using alcohol within BMIs. Specific behavior change components include personalized feedback, particularly normative feedback, in relation to students in the same university context. An alternative strategy, where feedback only is given to students on their blood alcohol concentration (BAC), has shown mixed evidence [8••].

Very brief interventions with personalized feedback and multicomponent interventions have been effectively adapted for digital use and, indeed, show promise for further development and research [9]. In fact, college students who engage in binge drinking (heavy episodic drinking (HED)) at least once a month have been found to prefer computerized methods of intervention [10]. Digital interventions for alcohol problems, primarily delivered via the Internet to a stationary computer, offer small but meaningful effects [11–15, 16••, 17]. When such digital interventions are evaluated in comparison with controls, they compare in effect size to face-to-face counseling. However, in direct comparisons between digital and face-to-face interventions, the latter have emerged as more effective [16••, 17]. Nonetheless, digital interventions hold the promise of much broader dissemination than face-to-face interventions and even small effect sizes can have considerable impact when interventions are widely available. Digital interventions in the form of automated mobile interventions, delivered over a phone network or through the Internet to handheld devices carried in everyday life, offer a new approach for delivering alcohol interventions to university students that could increase accessibility well beyond computer-based interventions. This specific topic has not, to our knowledge, previously been systematically reviewed.

Text messages are the most basic mode of automated mobile interventions and refer to brief electronic written messages transmitted via a mobile phone network. From an intervention point of view, such text messages can be used for monitoring and as a reminder system and can also include brief supportive messages to promote behavior change. When text messages are delivered to smartphones, they can include links to web pages with additional content and be combined with other computer-like capabilities such as GPS coordinates. Text messaging techniques have been reviewed for delivery of interventions in diverse settings, including the treatment of alcohol use disorders [18•, 19].

Automated telephony is often termed interactive voice response (IVR), a technology relying on a central computer programmed to administer incoming and outgoing calls over a phone network. Users access the central computer via their personal phone and listen to and navigate through content, responding to questions by voice recognition or touchtone technology. IVR is well-established technology for collecting data as well as for delivering interventions and has been reviewed in several areas of research including assessment and intervention in diverse populations [20, 21]. For the treatment of alcohol problems, studies with positive results have been conducted in such diverse populations as primary care patients [22] and paroled offenders [23].

In recent years, smartphones have offered individuals across the globe constant access to hand-held computers. Smartphone apps fill a multiplicity of functions and serve as virtual personal assistants on an everyday and, indeed, moment-to-moment level. Applications can be designed to function with or without an Internet connection. In the field of behavior change, apps are marketed as personal assistants for individual efforts towards improved health. For example, apps have been used to register weight for obesity control [24] and, in a guided version, to help users with behavioral activation in their own chosen valued direction [25]. In the area of alcohol consumption, research on smartphone apps is in its infancy. Available apps often have no therapeutic purpose and even provide incorrect information [26], although a qualitative study of user experiences indicated that alcohol-related apps could help users keep their alcohol use down [27]. A content analysis of behavior change techniques in alcohol apps found that most could be perceived as encouraging alcohol use [28•]. Techniques most often used included self-monitoring, information on negative consequences of alcohol use and positive consequences of abstinence, and personalized feedback [28•]. Although alcohol apps for smartphones are growing exponentially, the evidence for their effectiveness in reducing problematic alcohol use is lacking [29]. Whether apps can contribute to reducing hazardous or even harmful drinking is still an open question [30].

The purpose of this article is to present a review of recent scientific articles reporting on mobile phone-based interventions for college or university students. We have included interventions based on text messages, automated telephony, and smartphone apps.

## Method

The inclusion criteria for this review were that (a) the article explicitly studied members of the college and university population; (b) the topic of the article was a study of a behavioral/psychological intervention transmitted via mobile technology with an outcome directly related to alcohol consumption (e.g., quantity of alcohol consumed) or change in alcohol consumption (e.g., perceived change in alcohol consumption); and (c) the intervention was evaluated in comparison with a control group. Only English language articles were considered. Interventions primarily targeting other disorders but including alcohol-related disorders were excluded.

We conducted database searches in two stages. Our initial focus was on text message interventions and smartphone apps. To this end, we searched MEDLINE, PsycINFO, PsycARTICLES, PubMed, Scopus, and Web of Science using the following search words and Boolean operators: (smartphone OR mobile phone OR mobile device* OR sms OR short message service OR text messaging OR tablet OR iphone OR mobile technolog* OR smart phone OR ipad OR mhealth OR android OR windows) AND (alcohol OR substance) AND (college OR university OR student*). We restricted the search to a time frame from 1 January 2012 to 16 October 2015. The initial search returned 385 articles; 131 duplicate articles were removed, leaving 254 articles. Following this search, we decided to expand the initial criteria to include interventions based on automated telephony. This secondary search was identical to the procedure described above for text message and smartphone app interventions. We searched for articles including automated telephony interventions (IVR), adding the search terms IVR OR interactive voice response to the terms used in our original search; we identified 137 articles, of which 76 were unique and only 1 matched the inclusion criteria. This procedure was rerun using the term (automated telephon*) and yielded the same end result.

**Fig. 1:**
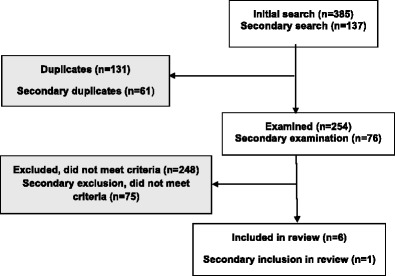
Flowchart of review process

Each article was screened for fulfillment of the above inclusion criteria. First, the title was scanned and if the article obviously covered topics and populations outside the scope of this article, it was excluded. This procedure was extended to the abstract if the title did not provide adequate information on whether to include or exclude the article. At a third level of scrutiny, if there was still some ambiguity with regard to the intervention/s or population studied, the article was read in full text. Articles obviously fitting the scope of this review on any of these levels were included and also read in full text. This resulted in exclusion of 248 and 75 articles, respectively. The remaining seven articles were included in this review. The search strategy is depicted in Fig. 1.

## Results

In this section, we describe the studies identified for the review, focusing on country, sample, intervention characteristics including delivery mode, content and duration, study conditions, follow-ups, outcome measures, and findings. Table 1 shows an overview of these studies.

**Table 1:** Overview of study sample characteristics, interventions, study conditions, outcome measures, and findings

Study citation	Country	Sample	Intervention	Study conditions	Follow-ups	Outcome measures	Summary of findings
Bendtsen and Bendtsen [31]	Sweden	*n* = 454 university students from semesters 2, 4, 6, and 8 female 48 %	Four types of fully automated push-based messages (food for thought messages, tasks, challenges, and reflective massages) delivered via text messages or via e-mail once a week (in total four messages per week) for 4 weeks. All messages included a direct link to the study’s home page providing information on safe drinking limits and a web-based single-session alcohol assessment	E_1_ = emailed messages four times/week *E*_2_ = sms four times/week No untreated control group Duration 4 weeks	Post-intervention (4 weeks from baseline)	SCAC	23 % of participants reported self-perceived change. *E*_1_ = *E*_2_
Mason et al. [32]	USA	*n* = 18 Hazardous drinking (≥8 on AUDIT) university students, psychology undergraduates Female 56 %	Four to six personalized text messages, based on MI principles with social network counseling, including reflections on peer risks and protection, were sent out daily for four consecutive days. Booster messages could be sent out on request	E = daily text messaging *C* = assessment only Duration 4 days	1 month	AUDIT, Quantity, DLO, MDLM, ADOLM	E = C
Moore et al. [33]	United Kingdom	*n* = 43 university students and *n* = 43 nonstudents, all >18 years with any alcohol consumption Female 33 %	One mobile text message delivering feedback on expenditures for alcohol in the past month, based on daily registration of alcohol consumption in the past month	E = daily text message drinking survey + one-time drinking expenditure feedback *C* = daily text message drinking survey Duration 2 months	1 month	DLD	E = C
Riordan et al. [34]	New Zealand	*n* = 295 University students, first year Female 55 %	EMA: text messages were sent out every night during orientation week with a health or social consequences of alcohol use, prompting students to apply the advice from the text messages in a real-world setting. The social messages were sent on nights with planed orientation events while health messages were sent on all other nights	*E* = Ecological Momentary Assessment (EMA)—(four messages during the orientation week + once a week during the first academic semester, asking to report number of standard alcohol units consumed) + Momentary Intervention (EMI)—(daily orientation week) *C* = EMA only (four messages during the orientation week + once a week during the first academic semester, asking to report number of standard alcohol units consumed) Duration university semester	EMA occasions during one university term	Quantity (orientation week), WD (semester)	*E* = C (*E* < *C* for women)
Andersson [35]	Sweden	*n* = 1678 Hazardous drinking (≥8 males/≥6 females on AUDIT) university students with cell phone numbers Female 41 %	The intervention was designed to reduce eBAC by providing personalized normative feedback and protective behavioral strategies, delivered either as a single intervention or as an intervention repeated at a 1-week interval. The intervention was delivered either by IVR to a mobile device or over the Internet to stationary computers	E_1_ = 1 IVR call, 1 week after recruitment E_2_ = 2 IVR calls, 1 and 2 weeks after recruitment E_1 + 2_ = 1 or 2 IVR calls *C* = assessment only Duration 1–2 weeks	E_1_ 5 weeks post intervention (6 weeks from baseline) E_2_ 4 weeks post intervention (6 weeks from baseline) *E*_1+2_ 4 or 5 weeks post-intervention (6 weeks from baseline) *C* 6 weeks from baseline	Primary: peak eBAC/week. Secondary: quantity, frequency, and mean eBAC/week, AUDIT	E_1_ < C AUDIT E_2_ < C peak eBAC, AUDIT E_1 + 2_ < C peak eBAC, AUDIT
Gajecki et al. [36]	Sweden	*n* = 1932 Hazardous drinking university students with smartphones Female 52 %	Intervention 1—Promillekoll: registration of one’s own alcohol consumption in real time, generating the user’s eBAC and providing information and feedback on harmful levels of eBAC (over 0.06 %). Several strategies for maintaining alcohol consumption below this limit were offered, as well as a warning if registered alcohol consumption would result in a harmful eBAC level. Intervention 2—PartyPlanner: eBAC level calculation, based on real-time registration of alcohol consumption; this can be used as a stand-alone tool and/or as a planner where the user can plan an event at which alcohol will be consumed in advance and see what the eBAC level would be throughout the event. Users who plan an event in advance and also log real-time drinking can visually compare the two events and evaluate whether their drinking plan held throughout the real event	E_1_ = smartphone app “Promillekoll”. Real-time eBAC E_2_ = smartphone app “PartyPlanner” C = assessment only Duration 7 weeks	Post-intervention (7 weeks from baseline)	Quantity, frequency, BOPW, average eBAC/week, peak eBAC/month	E_1_ > C, frequency E_2_ = C, all outcomes
Witkiewitz et al. [37]	USA	*n* = 94 Heavy drinking (≥5 drinks male, ≥ 4 drinks female at least once in past 2 weeks) and smoking (at least once a week) university students Female 28 %	BASICS-mobile: integration of some components of BASICS (that normally consist of personalized feedback about drinking behavior with components of cognitive-behavioral treatment, including education regarding the effects of alcohol on the brain and behavior, skills training, risk awareness, expectancy information, and suggestions for less risky drinking habits, as well as brainstorming alternatives to heavy drinking) and feedback about smoking and “urge-surfing,” a mindfulness-based approach, drawn from relapse prevention and mindfulness-based relapse prevention. The intervention was delivered in real-time via a smartphone for 14 days	E_1_ = BASICS mobile modules and daily EMA E_2_ = daily EMA C = assessment only Duration 14 days	1 month	DPDD, BOPW, YAAPST	E_1_ = more BASICS modules associated with reduced risk of drinking during intervention period E_1_ = E_2_ = C

Note: *BASICS* Brief Alcohol and Screening Intervention for College Students, *E* experimental condition, *C* control condition, *SCAC* self-perceived change in alcohol consumption, *Quantity* standard glasses consumed per week, *Frequency* drinking occasions per week, *BOPW* binge-drinking occasions per week, *eBAC* estimated blood alcohol concentration, *AUDIT* alcohol use disorder test, *DPW* drinks past week, *DLO* drinks last occasion, *MDLM* maximum drinks last month, *ADOLM* average drinks occasion last month, *DLD* drinks last day, *EMA* ecological momentary assessment, *EMI* ecological momentary intervention, *WD* weekend drinking, *DPDD* drinks per drinking day, *YAAPST* young adult alcohol problem screening test

In total, seven studies providing some measure of efficacy of interventions described in the articles were included in this review. Four of the six studies concerned text-message-based interventions [31–34], one was based on IVR [35], while two focused on web-based or native smartphone apps [36, 37]. Sample sizes varied greatly across the studies, ranging from 18 to 1932 students, with proportions of female study participants ranging from 28 to 56 %. Furthermore, intervention durations varied from one single occasion up to 2 months. Relatively short follow-up periods were described in all studies, from direct post-intervention in three of the studies [31, 35, 36], 1 month post-intervention in three additional studies [32, 33, 37], and about 3 months post-intervention in the remaining study [34].

### Interventions Delivered via Text Messages

Interventions delivered via text messages were described in four studies [32–34, 36]. Total sample sizes in these studies varied from 18 to 454 students, consisting of between 33 and 56 % females. Two interventions built on daily messages [32, 34], one included four messages per week [36], and one intervention consisted of sending out just one single message [33]. The duration of the interventions varied as well, from one single message occasion [33], a 4-day [32], 1-week [34], and 4-week period [36]. In two of the interventions, messages were personalized and tailored based on the recipients’ previously registered alcohol consumption and alcohol-related behavior [32, 33], while two interventions involved sending out untailored messages [34, 36]. Follow-up periods were relatively short for three of the four studies, with one conducting a post-intervention follow-up [36] and two implementing follow-up 1 month after the intervention period was finished [32, 33]. In the fourth study, the intervention was launched at the beginning of the university semester and follow-up took place at the end of the semester [34].

In terms of efficacy, one study [33] indicated nominal positive effects with regard to alcohol consumption, with the text-message-based intervention showing lower consumption for students in comparison to daily registration of alcohol consumption via text messages. However, due to low sample size, results were not statistically significant and are shown as such in Table 1 [33]. Another study [34] showed no overall differences regarding alcohol consumption between the text-message-based interventions and only registering alcohol consumption via the text messages, but positive effects of the intervention were found for female study participants. For the other two text message studies, one study [31] showed no differences in effect between the intervention being delivered via text messages or via e-mail and the other [32] showed no differences regarding alcohol consumption between the intervention and the assessment-only group but showed that the intervention was effective for increasing the motivation to change alcohol consumption; this result is not included in Table 1 due to our focus on the actual measures of change in alcohol consumption in this review.

### Interventions Delivered via IVR

Only one intervention using IVR, conducted by Andersson and colleagues [35], was identified. A total of 1678 university students with hazardous drinking, 41 % females, were randomized to an intervention delivered either by IVR to a mobile device or over the Internet to stationary computers or to an assessment-only control group. The intervention was designed to reduce peak blood alcohol concentrations and was delivered either as a single intervention or as an intervention repeated at a 1-week interval. The intervention included personalized normative feedback and protective behavioral strategies. Follow-up occurred 6 weeks after randomization, meaning 5 weeks after the single intervention or 4 weeks after the repeated intervention. Compared to controls, both the IVR and the Internet-based interventions led to a small but significant overall reduction in the primary outcome, peak blood alcohol concentrations. Personalized feedback was delivered to control group participants immediately after follow-up. The results indicated that the repeated IVR intervention might be required to achieve the identified effect.

### Interventions Delivered via Web-Based or Native Smartphone Applications

Two of the studies focused on mobile interventions using smartphone applications [36, 37]. Gajecki and colleagues [36] tested the effects of two web-based mobile phone brief intervention apps for hazardous alcohol use among university students (52 % women). The first app, Promillekoll, makes it possible for users to register their own alcohol consumption in real time, generating the user’s estimated blood alcohol concentration (eBAC). Further, the application provides information and feedback on harmful levels of eBAC (over 0.06 %), strategies to maintain alcohol consumption below that limit, and a warning if registered alcohol consumption is likely to result in a harmful eBAC level. The second app, PartyPlanner, includes the Promillekoll functions and also makes it possible for the user to plan an event where alcohol will be consumed in advance, as well as follow-up consumption after the event is concluded. PartyPlanner users can register what they plan to drink during the event, and the app will display the potential eBAC level throughout the event. Users who have planned an event in advance and then also logged real-time event drinking can also visually compare the two events and thus evaluate whether their drinking plan held throughout the real event. The effects of these two apps were evaluated among 1932 university students in a three-arm design with randomization to either one of the two apps or to an assessment-only control group. In total, 1364 students participated in follow-up 7 weeks after randomization. In comparison to the assessment-only control group, the results showed no effects for PartyPlanner, while Promillekoll users increased the number of drinking occasions in the 7 days preceding follow-up. Secondary analyses showed that the increase was valid only for male Promillekoll users [38].

Witkiewitz and colleagues [37] conducted a three-arm randomized controlled trial with 94 college students (28 % women) who both smoked and drank alcohol at least once a week and had at least one heavy drinking episode in the 2 weeks prior to recruitment. The purpose of the study was to investigate the effects of a mobile feedback intervention that targets both smoking and heavy episodic drinking. The intervention tested in this trial (Brief Alcohol Screening and Intervention for College Students (BASICS)-mobile) integrated some components of the BASICS that usually consists of personalized feedback about drinking behavior and feedback about smoking and “urge-surfing.” Study participants were randomized to receive the BASICS-Mobile intervention delivered in real time via a smartphone for 14 days, to daily monitoring via mobile assessment for 14 days, or to a minimal baseline assessment control group. Results from the 1-month follow-up showed that participants from both the BASICS-mobile intervention (*d =* 0.55) and mobile monitoring (*d* = 0.45) arms reduced the number of cigarettes per smoking day in comparison to control group participants. Furthermore, individuals assigned to BASICS-mobile who accessed a higher number of intervention modules were less likely to drink at all during the 14-day intervention period. This group had also reduced smoking at the 1-month follow-up period. Finally, at the 1-month follow-up, the BASIC-mobile intervention had no effect on reducing heavy episodic drinking or simultaneous smoking and alcohol use [37].

## Discussion

The results of this review can be summarized as inconclusive. The seven studies evaluated a variety of mobile phone-based interventions, including general; non-personalized text messages on the negative consequences of alcohol consumption or encouragement to reflect one’s own drinking; text messages, IVR, or apps providing personalized feedback, protective behavioral strategies, or social network counseling; and real-time alcohol consumption registration and estimation of blood alcohol concentration as well as planning of alcohol consumption in advance. Only the study evaluating single and repeated IVR interventions reported clear positive results for the outcome measuring alcohol consumption [35]. Three additional studies reported positive results, one concerning self-perceived change in alcohol use following a 4-week text message intervention [36], one indicating positive results for alcohol use for women but not for men following a semester-long text message intervention [34], and the third finding that greater use of intervention modules was associated with reduced drinking risk but only during the web-based app intervention period of 14 days [37]. A 4-day text message study [32] showed no differences in comparison to an assessment-only control group, and a 2-month study testing the addition of a one-time drinking expenditure feedback intervention to daily drinking registration via text messages showed no differences in comparison to only daily drinking registration [33]. Finally, a web-based app study offering access to one of two apps for 7 weeks showed no differences compared to an assessment-only control group for a web-based app and negative results for a native app, in comparison to the control group [36].

Interestingly, despite evidence that the number of apps related to alcohol use is in the thousands, only two studies evaluating apps among university students were identified. The pace of technological, market-adapted development is rapid, contrasting to the slower processes characterizing research-based development. Methodological innovations inspired by engineering indicate ways in which behavioral components could be sequentially tested for iterative, agile research-based development of optimized interventions [39–41], but citations of this type of methodology in the research literature are slow to come. Testing already existing interventions is one way to speed up the establishment of an evidence base, but the one study in our review that tested an existing native app showed the only negative result in this review [36]. Controlling for study participants’ use of publicly available apps during ongoing trials is a further challenge that needs to be addressed.

Closer attention to intervention content could be a way to optimize results. In this review, two studies used interventions based on prior research showing positive results in face-to-face and web-based interventions [35, 37]. The IVR intervention used two primary elements of the BASICS program—personalized feedback and teaching protective behavioral strategies targeting high consumption [41]. The web-based app showing positive results on drinking during the 14-day intervention period was also built on the BASICS program. The IVR intervention was simple, very brief, and focused only on one problematic behavior: binge drinking. The web-based app intervention included multiple modules and daily ecological momentary assessment and focused on two problematic behaviors: smoking and drinking. These contrasts invite the question of whether simplicity could be an advantage for obtaining positive results using mobile phone-based interventions. Could less really be more in this case?

All technical platforms in this review used a server programmed to communicate with handheld devices carried in many students’ everyday life, either over a phone line or over the Internet, offering students intervention content either in written or verbal form. Depending on user or researcher preferences, it is likely that any of the technical platforms covered in this review have the potential to be used for alcohol intervention, either as a stand-alone platform or in combination with other platforms. Given the high demand for digital interventions in the target population, multiple easily accessible platforms with a clear aim of reducing hazardous and harmful alcohol use are a necessary counterforce to smartphone applications that have alcohol-related entertainment as their main objective [26]. To reach beyond good intentions, researchers clearly need to now focus their attention on intervention content, rather than searching for a single technical solution that might solve the entire problem. Alternatively, research could focus on comparing specific combinations of interventions and technology, for example, personalized feedback via the Internet, text messages, IVR, or app compared to another intervention offered via the same channels. Research efforts seem likely to be more successful in relation to already established interventions that have the potential to be transferred to single or multiple, easy-to-reach intervention modules, in order to achieve efficacy on target outcomes.

## Conclusions

The area of research on mobile phone-based interventions targeting hazardous drinking among university students is a new one. An extensive search yielded a total of seven randomized outcome studies covering a time period of less than 3 years. Clearly, much research remains to be done. We identified one IVR study, four text message studies, and two app studies. The types of behavioral interventions used varied from ecological momentary assessment, self-monitoring at the user’s discretion, and personalized feedback to protective behavioral strategies. The control groups used for comparison were untreated in four of the seven studies, were offered minimal intervention in two of the studies, and absent in one of the studies. Outcome measures varied widely, obviating the possibility of comparing results between the studies and hampering efforts to draw meaningful conclusions from the studies reviewed.

Future research should focus on simple, brief interventions using proven methodology. The issue of control group conditions is ethically challenging, given that the study population engages in hazardous use of alcohol but is not seeking help. In studies of brief duration, control groups should optimally be untreated but given a relevant and effective minimally brief intervention (e.g., personalized feedback) after the final study follow-up. In studies of longer duration, control groups could be offered minimal intervention. Given the brief follow-up times reported, incoming research should follow participants over a longer period of time, post-intervention. Given that interventions can be perpetually available in app-based designs, the question of actual intervention use should be further explored, like the optimal dosing of an intervention (weekly, daily, or “just in time”) or its duration over time. Quick iterative studies that test behavioral components in innovative ways could offer a way forward (e.g., [41]). The huge potential of mobile phone technology for intervening to promote better alcohol-related health among university students beckons. The research field is young, and more robustly designed studies with positive outcomes could add the much-needed knowledge about interventions potentially contributing to meaningful, life-saving changes in individual behavior to the great benefit of students and those around them.
